# Epidemiology and Performances of Typhidot Immunoassay and Widal Slide Agglutination in the Diagnosis of Typhoid Fever in Febrile Patients in Bafoussam City, Cameroon: A Cross-Sectional Comparative Study

**DOI:** 10.1155/2024/6635067

**Published:** 2024-02-22

**Authors:** Aurelie Dahlia Yemeli Piankeu, Siméon Pierre Chegaing Fodouop, Michel Noubom, Emmanuel Boris Gomseu Djoumsie, Georges Ful Kuh, Donatien Gatsing

**Affiliations:** ^1^Department of Biochemistry, Research Unit of Microbiology and Antimicrobial Substances, Faculty of Science, University of Dschang, Dschang, Cameroon; ^2^Department of Biomedical Sciences, University of Ngaoundere, Ngaoundere, Cameroon; ^3^Department of Microbiology-Hematology and Immunology, Faculty of Medicine and Pharmaceutical Sciences, University of Dschang, Cameroon

## Abstract

**Background:**

Enteric fever is a great public health problem associated with significant illness and death in many endemic countries, and its clinical diagnosis is still daunting. The aim of this study was to determine the prevalence and risk factors of *S.* Typhi among febrile patients in Bafoussam and to evaluate the diagnostic performances of Widal and Typhidot tests.

**Methods:**

This was a cross-sectional study among 336 participants visiting three hospitals in Bafoussam from August 1, 2021, to November 31, 2021. Widal test, Typhidot assay, and stool culture were used to screen for salmonellosis with the help of a structured questionnaire.

**Results:**

The prevalence of *S.* Typhi and *S.* Paratyphi was found to be 62.85% and 37.14%, respectively. The overall prevalence of typhoid fever using stool culture was 20.86%. The significant risk factors associated with enteric fever were lack or insufficient knowledge of typhoid fever, poor hand hygiene, and anorexia. Typhidot immunoassay was more sensitive (100%) and specific (82.3%) than the Widal test. Both were analytically inferior to stool culture.

**Conclusions:**

High prevalence of typhoid fever (20.86%) was observed which was largely associated with lack or insufficient knowledge of typhoid fever, poor hygiene measure, and anorexia as risk factors. The performances of the Widal and Typhidot test against a stool culture were inferior but with Typhidot better than the Widal slide agglutination.

## 1. Introduction

Typhoid or enteric fever is a systemic, bacterial, potentially severe infection linked to poor hygiene practices. It is caused by *Salmonella* Typhi, a Gram-negative bacterium. It has become uncommon in developed countries due to progress in hygiene and improved water supply conditions [1, 2]. However, it is one of the main causes of hospital admissions in developing countries [3, 4]. The disease is transmitted via the fecal-oral route from Salmonella contaminated food or dirty water [5]. The main and common symptoms of the disease include malaise, fever, vomiting, constipation, splenomegaly, and hepatomegaly [6]. The disease can lead to serious complications such as internal bleeding and intestinal perforation [7]. However, despite the decline in morbidity and mortality from typhoid fever in developed countries, it remains one of the main major public health problems in developing countries, particularly in Central Africa where it is endemic [2].

The real combat in the treatment of typhoid fever lies in its misdiagnosis in the clinical setting due to overlapping symptoms with other common infections such as malaria, dengue, and viral diseases [8] Accurate and rapid diagnosis of typhoid fever depends on isolating the pathogen and identifying potential carriers of the infection [9]. Detection of *S.* Typhi can be done from many body fluids and biomaterials such as blood, stool, urine, bone marrow, rose spot extracts, duodenum, and aspirates [10]. Blood, stool, and urine cultures are the best diagnostic methods, but they are very costly techniques and require equipment and qualified personnel who are often lacking in many clinical training institutions in Cameroon. The different methods employed to diagnose typhoid fever are clinical signs and symptoms, the use of serological markers (Widal test), bacterial culture (stool culture and blood culture), antibody detection (Typhidot), and molecular methods (amplification of DNA by PCR) [11]. Blood culture and stool culture are the two most specific tests in the diagnosis of typhoid fever [12], the gold standard for diagnosing typhoid fever is blood culture, but it is expensive for patients, time-consuming, and in remote rural settings, culture facilities may be unavailable [13]. Stool culture could also be used where blood culture is inaccessible because of a strong agreement between blood and stool culture for diagnosing typhoid fever [14]. Widal slide agglutination test is very simple and affordable to perform and requires minimal training and equipment and has been used in the diagnosis of typhoid fever for a long time in Cameroon. It remains a very common and good serological test with a moderate specificity, but its sensitivity in the diagnosis of typhoid fever is hard to interpret for several reasons: delayed time between infection and antibody production, cross-reactivities, and the long stay of target serum antibodies after treatment with lack of active disease [15]. Typhidot immunoassay is a rapid serological assay for the diagnosis of enteric fever and can be used as an alternative to Widal test due to its better sensitivity and specificity [16]. However, Typhidot is not suitable to replace blood culture [8]. Can Typhidot be better than stool culture is the question we ask ourselves? Stool culture is still dominantly used as a confirmatory test for typhoid fever in most sub-Saharan clinical facilities owing to its low cost relative to hemoculture. This study was carried out to determine the prevalence and risk factors of *S.* Typhi among patients in Bafoussam and to evaluate the diagnostic performances of Widal test and Typhidot immunoassay vis-à-vis stool culture among febrile patients in the city of Bafoussam.

## 2. Methods

### 2.1. Study Area and Period

**Figure 1:**
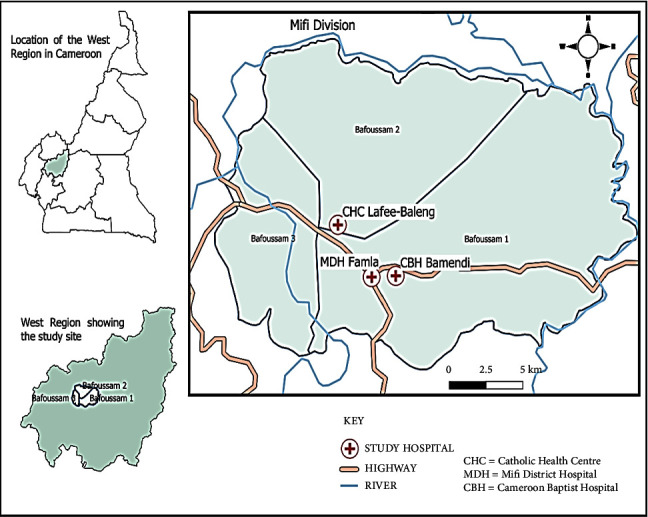
Sample collection location sites in West Region (turquoise circles represent study sites).

The city of Bafoussam is located on the Bamileke plateau at 1420 m altitude and 5° 28′ north latitude. The Department of Mifi is one of the six departments of the West Region of Cameroon with Bafoussam as its capital. This department is divided into three districts as follows: Bafoussam 1, Bafoussam 2, and Bafoussam 3 (Figure 1). Its population constitutes 465,000 inhabitants with an area of 402 km^2^ with a long rainy season and a short dry season. Precipitation there varies around 1717.7 mm, and temperatures vary around 13.6°C to 25.35°C. The population's livelihood is mainly on agriculture, livestock, and trade. The main agricultural products are corn, beans, potatoes, carrots, cabbage, banana, and plantains. The main animals raised are chickens and pigs.

This study took place during the period from August 1, 2021, to November 31, 2021.

### 2.2. Study Design and Population

A cross-sectional study was conducted in three hospitals in the city of Bafoussam (Bingo Cameroon Baptist Hospital of Bamendi; Mifi District Hospital and Catholic Health Center of Lafee-Baleng) on patients with symptoms of gastroenteritis or typhoid fever. All eligible participants (temperature >37.5°C at inclusion) who gave their written informed consent were recruited and attributed a unique study code. This code was used for all samples collected and different for each patient. A questionnaire containing demographic, clinical, and lifestyle information was administered to the patient. Three hundred and thirty-six patients were recruited based on the criteria listed in Table 1.

### 2.3. Assay Methods

#### 2.3.1. Blood Collection

Using a sterile syringe and needle, 3 mL of venous blood was drawn from each patient's forearm into a dry tube prelabeled with an anonymized patient code. The analysis of the blood samples was done by a qualified laboratory technician.

#### 2.3.2. Test Procedure for the Typhidot Immunoassay Test

Typhidot test (Medsource ozone bio medicals, Faridabad, India) is an immunochromatographic assay designed for simultaneous detection and differentiation of specific IgM and IgG antibodies against *S.* Typhi in human serum. The diagnostic test cassette consists of two components: an IgG component and an IgM component. The (G) line region is precoated with reagents for the detection of anti-*S.* Typhi (IgG) and the (M) line region is precoated with monoclonal antihuman IgM for detection of anti-*Salmonella* Typhi (IgM). The (C) line corresponds to the negative control. During testing, serum is dispensed into the sample well and antibodies in serum bind to typhoid antigen conjugates impregnated in the reagent area if the serum contains antityphoid antibodies.

Using a sterile syringe and needle, 3 mL of venous blood was collected from each patient's forearm into a dry tube prelabeled with an anonymized patient code. The blood specimens were then spun at 3000 rpm for 5 min using a laboratory centrifuge. Using the provided pipette, one drop of serum was added to the test well on the test cassette followed by one drop of buffer. The setup was allowed for 15 minutes for migration across the membrane and color development in the result windows. A positive IgM was interpreted clinically as acute typhoidal disease, while IgM and IgG positive were taken as acute typhoidal fever disease in the middle stage of infection. IgG positive band indicated a chronic carrier or previous infection or reinfection. A colored control line (C) must always appear in case of a negative or a positive result. Its absence indicates invalid test results.

#### 2.3.3. Widal Slide Agglutination Test Procedure

Widal agglutination test was performed using febrile antigen kits of *Salmonella typhi* (Chromatest Febrile Antigens kits, linear chemicals, Barcelona, Spain). Tests were done by laboratory professionals who were blinded to the study and were based on the manufacturer's guidelines. The slide agglutination test was used as a screening test for the presence of anti-TO and anti-TH antibodies in the participant's serum. Then, a drop of serum was placed on the test card for both O and H antigens. A drop of *S.* typhi O and H antigens was added on a drop of serum on card and rotated at 100 rpm for 2 minutes on an orbital shaker and reported as reactive or nonreactive. Appreciation of reactive or nonreactive was done by the comparison of the test sample to negative control. Reactive Widal test was determined by the formation of visible agglutination reaction of *S.* typhi antigen with the patient's serum antibody on the testing card. Nonreactive test was determined by the absence of a visible agglutination reaction of *S.* typhi antigen.

#### 2.3.4. Stool Sample Collection and Culture

In typhoid fever, coprocultures are usually positive from the second week of the infection. Stool cultures were performed at the Famla laboratory. Approximately 4 g of fresh stool were collected from each patient suspected to have *Salmonella* infections for the coproculture test after a brief training on how to collect the sample using a sterile wide-mouthed and aseptic transparent container. Materials were provided to each of the participants for stool collection. After collection, the stool was emulsified in 3 ml normal saline and 1 ml of this inoculum was diluted with 9 ml of freshly prepared Selenite F Broth (Oxoid CM0395B and LP0121A) in prelabeled tubes and incubated at 37°C for 24 hours aerobically in an incubator for bacterial growth. A loop full of inoculum from 3 ml normal saline was inoculated by the streaking method onto *Salmonella Shigella* selective agar (SSA) (Neogen, Marshfield, United States) and MacConkey agar plates (McConkey and Co, Puyallup St, United States). Incubation of all culture plates were carried out at 37°C for 24 hours. Isolation of *S.* typhi in stool culture indicated an infection. In the absence of growth, the culture was considered negative and subcultures were repeated for one week. Isolates were confirmed to be *S.* typhi and *S.* Paratyphi by API20E gallery (bioMerieux, Marcy-l'Étoile France) and by agglutination with *Salmonella* agglutinating sera [17]. The qualified laboratory personnel who performed coprocultures were blinded to the procedure and results of the Widal, Typhidot, and stool culture tests.

### 2.4. Quality Control

All the instruments used for sample processing were checked every morning for proper functioning, and standard procedures were followed during processing of each sample.

### 2.5. Ethical Approval

Cameroon National Committee for Ethics in Human Health (Ref no 2022/08/128/CE/CNERSH/SP) approved the experimental procedures and protocols used in this study. The concept of the study was explained to the participants, and written informed consent was obtained from all participants before enrolment. A designed questionnaire was administered to each participant to collect clinical symptoms, hygienic, behavioral, and demographic information.

## 3. Statistical Analyses

The sample size was calculated based on the nomogram published by Carley et al. (2003). Based on this nomogram, we calculated the sample size by using the following criteria: an estimated prevalence of typhoid fever of 31%, a sensitivity plot at alpha = 0.05, a confidence limit of 95%, and a type 1 error of 0.05. We obtained for this study an estimated sample of 336 patients. Collected data were entered into Excel and exported to SPSS (Statistical Package for Social Science) version 24.0 for further analysis. The chi-squared (*X*^2^) test was used to check the statistical significance, and significance was tested at *p* ≤ 0.05. The sensitivity, specificity, and predictive values were calculated based on standard formulae, while the area under the receiver operating curve was used to estimate the diagnostic accuracy of the Widal and Typhidot tests. Kappa test was used to assess the agreement between the tests. The test accuracy, sensitivity, specificity, positive predictive value, and negative predictive value were compared to Typhidot immunoassay and Widal slide agglutination test against stool culture. Logistic regression analysis was used to determine associations of sociodemographic, hygienic, behavioral, and clinical characteristics with typhoid fever.

## 4. Results

**Figure 2:**
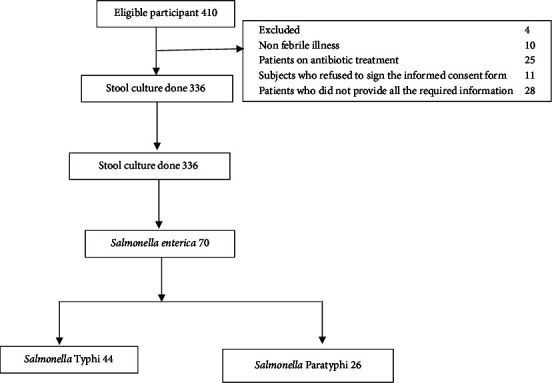
Flow diagram of febrile patients with positive stool culture in Bafoussam, Cameroon.

Of the 410 patients admitted and screened for clinical features of febrile illness or history of fever, 74 were excluded from the study, viz., 10 were nonfebrile illness patients, 25 were patients on antibiotic treatment, 11 were patients who refused to sign the informed consent form, and 28 were patients who did not provide all the required information. The stool cultures were collected from 336 patients who met the inclusion criteria (Figure 2). Out of the 336 patients included, 70 were positive for *Salmonella enterica* in whom 44 for *Salmonella* typhi and 24 for *Salmonella* typhi infection.

### 4.1. Sociodemographic Characteristics of Study Participants

A total of 336 patients presenting with the signs and symptoms of typhoid fever were received for consultation in three hospitals in the city of Bafoussam: Bingo Cameroon Baptist Hospital of Bamendi, MiFi District Hospital, and Catholic Health Center of Lafee-Baleng. Women were the most represented in this study [58.33% (196/336)] (Table 2). The age of the participants ranged from 1 to 60 years old with an average age of 25.20 ± 16.77 years, and the age groups represented were [1–10] years (58.03% (195/336)), [11–20] (17.55% (59/336)), [21–30] (9.82% (33/336)), [31–40] (8.33% (28/336)), [41–50] (3.86% (13/336)), and [51–60] (2.38% (8/336)). Concerning their level of educational attainment, 45.67% of the participants had a secondary level of study and 7.14% (24/336) were illiterate. Regarding marital status, 66.66% were single and 18.15% were married (Table 2). Profession wise, students were the most represented at 64.88% followed by private sector workers (27.97%), civil servants (3.57), and housewives (3.50) who were less represented. Participants living in urban areas were the most represented (64.28%) unlike those living in rural areas (35.71%) (Table 2).

### 4.2. Distribution of the Prevalence of Typhoid Fever in the Study Population

Table 3 presents the prevalence of typhoid fever according to sociodemographic characteristics and reveals a significant difference (*p*=0.01) between occupation and *Salmonella* typhi and *S.* Paratyphi infection.

Indeed, there was a higher prevalence of *S.* typhi and *S.* Paratyphi among private sector workers. Characteristics such as sex, age, and level of education were not significantly related to *S.* typhi and *S.* Paratyphoid infections. This table also shows the prevalence of typhoid fever according to lifestyle. We noted a significant difference between the level of knowledge of typhoid fever and infection with *S.* typhi and *S.* Paratyphi (*p*=0.07). The other parameters such as knowledge on environment, routes of transmission, hygiene measures taken against typhoid, type of drinking water used, and measures taken to make water drinkable were not linked to the infections of *S.* typhi and *S.* Paratyphi.

Regarding the signs and symptoms, a significant difference was observed between vomiting, fever, constipation, anorexia, and infection with *S.* typhi and *S.* Paratyphi with a *p* value of 0.01, 0.03, 0.008, and 0.02, respectively (Table 4). Parameters such as headache, body aches, chills, diarrhea, asthenia, type of stool, and abdominal pain did not show a significant difference with *S.* typhi and *S.* Paratyphi infections (Table 3).

### 4.3. *Salmonella* Serotypes

**Figure 3:**
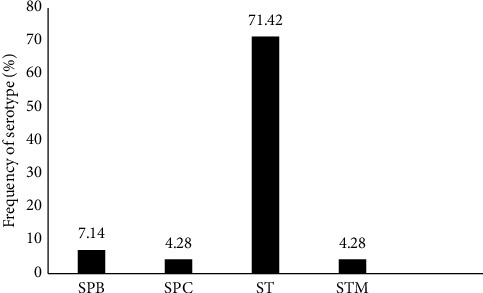
The different serotypes of *Salmonella*. SPA: *Salmonella* Paratyphi A; SPB: *Salmonella* Paratyphi B; SPC: *Salmonella* Paratyphi C; ST: *Salmonella* typhi; STM: *Salmonella* Typhimirium.

Figure 3 illustrates the different *Salmonella* serotypes isolated from febrile patients. API 20E Gallery used identified five main types of *Salmonella* strains including *Salmonella* typhi, *Salmonella* Paratyphi A, *Salmonella* Paratyphi B, *Salmonella* Paratyphi C, and *Salmonella typhimirium*. The *S.* typhi strain was the most represented (71.42%) followed by the *S.* Paratyphi A strain (12.85%), *S.* Paratyphi C (4.28%), and S. typhimirium (4.28%) strains which were the least represented.

### 4.4. Associated Risk Factors of Typhoid Fever

Sociodemographic parameters such as gender, age groups, level of education, marital status, occupation, and residence were used to determine the risk factors. A logistic regression analysis was performed to investigate the association between these factors and typhoid fever. None of the assessed sociodemographic parameters were found to be significantly associated with typhoid fever (Table 4).

### 4.5. Lifestyle and Clinical Symptoms

#### 4.5.1. Lifestyle

Knowledge of typhoid fever was significantly associated with typhoid fever (*p*=0.043). Indeed, the logistic regression showed that the absence or insufficient knowledge of typhoid fever in patients exposed them to typhoid fever (50%) compared to those with knowledge (20.12%) (Table 5). Lack of knowledge of typhoid fever strongly predicted exposure to typhoid fever (OR = 5.243, 95% CI: 1.052–26.119). Regarding hygiene measures against typhoid fever, multivariate logistic regression showed that patients who did not wash hands after bowel movements were positively associated with typhoid fever (3.740, 95% CI: 1.515–9.234).

#### 4.5.2. Clinical Symptoms

The most recurrent symptoms of typhoid fever reported by study participants were headache (21.12%) followed by vomiting (18.51%), muscular pains (20.51%), fever (20.52%) frisson (20.99%), diarrhea (23.55%), constipation (19.93%), asthenia (23.77%), anorexia (16.83%), and abdominal pain (20.60%). Multivariable logistic regression showed that anorexia was positively associated with typhoid fever (*p* ≤ 0.001) (Table6). Patients without anorexia (OR = 1) were greatly afflicted with typhoid fever compared to those with anorexia (OR = 7.703, 95% CI: 3.507–16.919). Headache, vomiting, muscular pain, fever, frisson, diarrhea, constipation, asthenia, type of stool, and abdominal pain were not found to be associated with typhoid fever (Table 7).

## 5. Comparison of Typhidot, Widal Slide Agglutination Test with Stool Culture in Diagnosis

We compared Widal and Typhidot in their ability to diagnose typhoid fever relative to stool culture. A sensitivity (100%), specificity (82.3%), predictive value of the positive (59.8%) and negative test (100%), and accuracy (86.0%) were observed for Typhidot-IgM and 98.5%, 66.1%, 43.4%, 99.4%, and 72.9%, respectively, noted for the Widal slide agglutination test. The accordance between Typhidot-IgM and Widal slide agglutination test was analyzed by Cohen's kappa test. However, there was moderate agreement between the Widal slide agglutination test and stool culture (kappa: 0.433, CI 95% 0.358–0.522) and a substantial agreement between the Typhidot-IgM test and stool culture (kappa: 0.660, CI 95% 0.575–0.744). The percentage agreements were 70.26% and 51.54% for Typhidot-IgM and Widal slide agglutination test, respectively (Table 8).

### 5.1. Antibody Screening with Typhidot Assay

Out of 336 samples, 92 (27.38%) were IgM and IgG positive, 73 (21.72%) were IgM positive and IgG negative, 60 (17.85%) were IgM and IgG negative, 117 (34.82%) were IgM positive, and 159 (47.32%) were IgG positive. The highest cases of typhoid fever were reported with IgG positive (Table 6).

### 5.2. Prevalence of Typhoid Fever Based on Three Diagnostic Methods in the Study Population

Table 9 summarizes the differences in prevalence estimates by the three tests. Out of the 336 participants in this study, 159 were positive for typhoid fever with the Widal slide agglutination test with an antibody titer of 1 : 80 for both “O” and “H” antigens being taken as cutoff point values indicative of a recent typhoid infection. This gives a prevalence of 47.32% and 26.78% (*n* = 90) who was negative for typhoid fever when stool culture was used as the reference diagnostic tool. With the Typhidot-IgM detection method, 117 were positive with a prevalence of 34.82% and 13.98% (*n* = 47) who were negative for typhoid fever when stool culture was used as the reference diagnostic tool. The difference in estimating the prevalence of typhoid fever was significant (*p* < 0.0001) when comparing the four diagnostic methods.

Also, the Widal test identifies 47.32% of febrile patients suspected of typhoid fever as positive, whereas only 26.86% of these patients were positive for the Typhidot immunoassay. The low accuracy of the Widal agglutination test compared to Typhidot could be explained by exposure to crossreacting antigens in febrile infections other than *Salmonella* typhi. Typhidot test identifies 34.82% of febrile patients suspected of typhoid as positive, whereas only 13.96% of these patients were positive for the reference test.

## 6. Discussion

Typhoid fever continues to be a menacing cause of illness and death worldwide, especially in countries with insufficient resources such as Cameroon. The clinical diagnosis of enteric fever is not easy to arrive at owing to presentation of signs and symptoms that may overlap with febrile illnesses in regions where typhoid is endemic [18]. This work is the first study done on the association of risk factors to typhoid fever in this part of Cameroon.

In this study, a total of 336 samples were taken from patients attending three main hospitals of the West region of Cameroon, with the mean age of 25.20 ± 16.77 years. The prevalence of typhoid fever among febrile patients in the three different health centers of Bafoussam with stool culture was 20.86% indeed high. This finding was similar to that obtained in Buea-Cameroon (21%) [19] but higher than studies conducted in Ethiopia (5%) [20] and 15.3% [21] and in Indonesia (15.5%) and Lalitpur 4.1% [18]. This high prevalence of typhoid fever observed in this study can be attributed to the nonspecific clinical symptoms that can overlap with febrile illnesses in regions where typhoid is endemic [22]. Lack or insufficient knowledge of typhoid fever and hygiene measures such as not washing hands after bowel movements and anorexia may explain this prevalence rate. Participants who lacked or had insufficient knowledge of typhoid fever were found to be significantly more infected by typhoid fever pathogens. This might be explained by their level of education where primary and secondary young students were more infected and had poor practices of the hygiene measures against typhoid. This suggests that vital information on the knowledge and prevention of the disease is needed in the health districts in Cameroon via periodic sensitization seminars.

Talking of the hygiene measures against typhoid fever, a statistically significant association was observed between washing hands after bowel movements and typhoid or paratyphoid infection positivity. We noted that most participants who tested positive for typhoid failed to wash their hands regularly. Thus, lack of hand hygiene is a great risk factor to contracting and transmitting enteric infections. This notion is buttressed by an earlier report which demonstrated that those who prepared food and operated restaurants did not perform hand hygiene after toilet use and constituted a transmission risk factor to those who visited to eat, thus establishing a chain of infectivity for typhoid fever [23]. Our findings are also endorsed by the dramatic story of the typhoid fever carrier cook, Mary Malon, who inadvertently contaminated 7 out of 8 families during her professional cooking career [24]. All these observations and reports suggest that hand washing with soap after bowel movements is very important to prevent typhoid fever [25]. Analysis by symptoms and clinical findings showed anorexia was associated to typhoid and contributed to the diagnosis in patients with typhoid fever. This result is in accordance with the study carried out by [26] in Douala-Cameroon.

The diagnosis of typhoid has relied on the isolation of *Salmonella* species from blood, feces, urine, and bone marrow, with the isolation rate varying from 30% to 70%. Isolation of the causative agent by culture has remained the gold standard for diagnosis of typhoid fever [8]. However, it is well recognized that facilities for culture are not readily available in low-income or resource-limited countries. The Widal test, commonly employed in our setting, has been available for decades and can be performed using venous blood. Unfortunately, the assay has low sensitivity and specificity especially in endemic zones and optimally requires comparison of samples drawn at the acute and convalescent stage of illness [27]. In a developing country like Cameroon, the Widal test has been used extensively in the serodiagnosis of typhoid fever and stool culture as confirmatory test. Though blood and bone marrow cultures and PCR are considered as gold standard or reference [8], we could not afford to incorporate this standard. Nonetheless, stool culture and blood culture have been reported to have a strong agreement [14]. Our results of the Widal test showed that out of 336 samples, 159 were positive for *S.* typhi and *S.* Paratyphi with a prevalence of 47.32%. This prevalence was 34.82% (117 cases) with Typhidot. These results revealed disparities in the analytic performances of the Widal slide agglutination and the Typhidot technique. When compared to the stool culture, the Widal test had sensitivity, specificity, predictive values of the positive and negative tests, and accuracy of 98.5%, 66.1%, 43.4%, 99.4%, and 72.9%, respectively. Whereas, the Typhidot assay had a sensitivity, specificity, predictive values of the positive and negative tests, and accuracy of 100%, 82.3%, 59.8%, 100%, and 86.0%, respectively. These results indicate that the Widal test performs poorly compared to the Typhidot test, taking stool culture and fever within three days preceding hospital attendance as referent. These results are consistent with those in India [13–28]. Low sensitivity for the Widal agglutination test can be related to the data collection time. The Widal test correctly identifies many patients who are uninfected with *S.* typhi and *S.* Paratyphi (high specificity), while only about 26.48% of uninfected patients will be wrongly classified as having typhoid fever (false positives); meanwhile, they are actually uninfected. So, a negative Widal agglutination test result has a good predictive value for the absence of typhoid fever, but a positive result would have a low predictive value for the presence of disease [29]. Also, the Widal test identifies 47.32% of febrile patients suspected of typhoid fever as positive, whereas only 26.86% of these patients were positive for the Typhidot immunoassay. The low accuracy of the Widal agglutination test compared to Typhidot could be explained by exposure to crossreacting antigens in febrile infections other than *Salmonella typhi* [30]. The Typhidot test identifies 34.82% of febrile patients suspected of typhoid as positive, whereas only 13.96% of these patients were positive for the stool culture reference test. This observation could be due to early sampling before antibodies become detectable in blood as was reported in one study [13]. Moreover, in this study, Typhidot-IgM and stool culture analyses agree more by 70.26% than with the Widal agglutination test. A recent report has shown that stool antigen RDT concurs by 86.7% with stool culture [21]. This suggests that antibody and antigen detection by RDTs is credible and needs optimization of the technology to yield more sensitive results. Furthermore, stool culture, though with its own shortcomings, still stands as a better reference test in resource-deficient and low-income countries where blood culture and PCR would be very expensive for the local population.

Our study had some limitations. This study regrets meeting up with the use of the reference standard (blood culture or PCR) owing to lack of funding or limited resources. The low number of participants which could greatly affect the statistical power of the study was not attained. Responses provided in the questionnaire for age group below 12 years were given by parents and guardians who could introduce recall bias in the study.

## 7. Conclusion

The overall prevalence of typhoid fever based on stool culture was 20.86%, and this was associated with lack or insufficient knowledge of typhoid fever, absence of hand washing after toilet use, and anorexia as clinical symptoms. The performance of the Widal slide agglutination test and Typhidot immunoassay against a stool culture showed analytic inferiority. Typhidot immunoassay was more sensitive (100%) and specific (82.3%) than the Widal test and is a better alternative in the diagnosis of typhoid fever. There was a substantial agreement between the Typhidot-IgM technique and stool culture. Stool culture remains a better assay than Typhidot and a better reference assay in resource-limited settings. Thus, we recommend the use of Typhidot immunoassay in the detection of enteric infections in Cameroon's healthcare institutions and the great reduction of the use of Widal assay where appropriate.

## Figures and Tables

**Table 1:** Summary of study participant recruitment criteria.

Inclusion criteria^a^ (*N* = 410)	Noninclusion criteria (49)^b^	Exclusion criteria^c^ (*n* = 25)
(i) Patients with fever, temperature ≥37.50 C		(i) Subjects with hemolyzed blood
(ii) Subjects of at least one year old, regardless of sex or ethnicity, who have given a favorable opinion to participate freely in the study, or parental consents	(i) Patients on antibiotic treatment (ii) Subjects who refused to sign the informed consent form	(ii) Patients who did not provide all the required information (iii) Febrile subjects with malaria and respiratory infections

^a^Inclusion criteria are characteristics that the prospective subjects must have if they are to be included in the study. ^b^Noninclusion criteria are characteristics that subjects must not have if they are to be included in the study. ^c^Exclusion criteria are features that exclude subjects from the study in progress.

**Table 2:** Sociodemographic characteristics and frequency distribution of the study population.

Characteristics	Number (*n* = 336)	Frequency (%)
*Gender*		
Female	196	58.33
Male	140	41.66

*Age groups (years)*		
[1–10]	195	58.03
[11–20]	59	17.55
[21–30]	33	9.82
[31–40]	28	8.33
[41–50]	13	3.86
[51–60]	8	2.38

*Level of education*		
Primary	137	40.77
Secondary	153	45.67
Higher	22	6.56
No level (illiterate)	24	7.14

*Marital status*		
Married	61	18.15
Single	224	66.66
Divorced	32	9.52
Widow	19	5.65

*Occupation*		
Student	218	64.88
Private	94	27.97
Civil servant	12	3.57
Housewife	12	3.5

*Residence*		
Urban area	216	64.28
Rural area	120	35.71

^*∗*^The denominator used to calculate the frequency in column three is 336.

**Table 3:** Prevalence of typhoid fever by sociodemographic characteristics, lifestyle, and signs and symptoms in the study population.

Characteristics	Number (*N* = 336)	*Salmonella* Typhi positive (*n* = 44)	*Salmonella* Paratyphi positive (*n* = 26)	*p* value (*X*^2^)
Sociodemographic characteristics				

*Gender*				
Female	196 (58.33)	34 (77.27)	19 (73.07)	0.76 (0.09)
Male	140 (41.66)	10 (22.72)	7 (26.92)	

*Age groups (years)*				
[1–10]	195 (58.03)	27 (61.36)	14 (53.84)	
[11–20]	59 (17.55)	7 (15.90)	6 (23.07)	
[21–30]	33 (9.82)	5 (11.36)	3 (11.53)	0.62 (3.50)
[31–40]	28 (8.33)	2 (4.54)	3 (11.53)	
[41–50]	13 (3.86)	2 (4.54)	0 (0)	
[51–60]	8 (2.38)	1 (2.27)	0 (0)	

*Level of education*				
Primary	137 (40.77)	20 (45.45)	10 (38.46)	
Secondary	153 (45.53)	19 (43.18)	11 (42.30)	
Higher	22 (6.54)	3 (6.81)	3 (11.53)	0.96 (0.29)
No level	24 (7.14)	2 (4.54)	2 (7.69)	

*Marital status*				
Married	61 (18.15)	7 (15.90)	9 (34.61)	0.34 (3.55)
Single	224 (66.66)	31 (70.45)	15 (57.69)	
Divorced	32 (9.52)	4 (9.09)	1 (3.84)	
Widow	19 (5.65)	2 (4.54)	1 (3.84)	

*Occupation*				
Student	218 (64.88)	28 (63.63)	7 (26.92)	
Private	94 (27.97)	15 (34.09)	15 (57.69)	
Civil servant	12 (3.57)	0 (0)	2 (7.69)	**0.01** (11.03)
Housewife	12 (3.57)	1 (2.27)	2 (7.69)	

*Residence*				
Urban area	216 (64.28)	28 (63.63)	18 (69.23)	0.63 (0.22)
Rural area	120 (35.71)	16 (36.36)	8 (30.76)	

Life style				

*Do you know about typhoid fever?*				
Yes	328 (97.61)	44 (100)	22 (84.61)	
No	8 (2.38)	0 (0)	4 (15.38)	**0.007** (7.17)

*If yes, state knowledge source*				
Hospital	69 (20.53)	11 (25.00)	8 (30.76)	
School	117 (34.82)	16 (36.36)	8 (30.76)	
Media	27 (8.03)	4 (9.09)	2 (7.69)	0.94 (0.39)
Street	123 (36.60)	13 (29.54)	8 (30.76)	

*Typhoid transmission route*				
Soiled food	174 (51.78)	22 (50.00)	16 (61.53)	0.34 (4.49)
Blood route	13 (3.86)	2 (4.54)	0 (0)	
Contact with a sick person	41 (12.2)	5 (11.36)	5 (19.23)	
Dirty water	96 (28.57)	13 (29.54)	4 (15.38)	
From mother to child	12 (3.57)	2 (4.54)	1 (3.84)	

*Hygiene measure against typhoid*				
Do not washing hand after bowel movements	86 (25.59)	8 (18.18)	3 (11.53)	0.32 (4.62)
Hand washing before and after meal	46 (13.69)	7 (15.90)	3 (11.53)	
Washing fruits and vegetables before consumption	82 (24.4)	7 (15.90)	10 (38.46)	
Hand washing after returning from public places	70 (20.83)	10 (22.72)	5 (19.23)	
Hand washing after greeting a loved one	52 (15.47)	12 (27.27)	5 (19.23)	

*Drinking water used*				
Camwater	82 (24.40)	11 (25.00)	11 (42.30)	0.52 (4.15)
Rain water	6 (1.78)	1 (2.27)	0 (0)	
Well	21 (6.25)	6 (13.63)	1 (3.84)	
River	14 (4.16)	3 (6.81)	1 (3.84)	
Drilled holes (bore holes)	200 (59.52)	22 (50.00)	12 (46.15)	
Mineral	13 (3.86)	1 (2.27)	1 (3.84)	

*Hygiene measure used to make water safe to drink*				
Chlorination	48 (14.28)	5 (11.36)	3 (11.53)	
Filtration	84 (25.00)	6 (13.63)	7 (26.92)	
Boiling	13 (3.86)	2 (4.54)	0 (0)	0.53 (3.15)
Decanting	21 (6.25)	1 (2.27)	1 (3.84)	
None	170 (50.59)	30 (68.18)	15 (57.69)	

Signs and symptoms				

*Headaches*				
Yes	232 (69.04)	32 (72.72)	17 (65.38)	
No	104 (30.95)	12 (27.27)	9 (34.61)	0.51 (0.42)

*Vomiting*				
Yes	189 (56.25)	27 (61.36)	8 (30.76)	
No	147 (43.75)	17 (38.63)	18 (69.23)	**0.01** (6.11)

*Aches and pain*				
Yes	234 (69.64)	31 (70.45)	17 (65.38)	
No	102 (30.35)	13 (29.54)	9 (34.61)	0.65 (0.19)

*Fever*				
Yes	268 (79.76)	38 (86.36)	17 (65.38)	**0.03** (4.27)
No	68 (20.23)	6 (13.63)	9 (34.61)	

*Shivering*				
Yes	281 (83.63)	36 (81.81)	23 (88.46)	0.46 (0.54)
No	55 (16.36)	8 (18.18)	3 (11.53)	

*Diarrhea*				
Yes	208 (61.90)	28 (63.63)	21 (80.76)	0.13 (2.28)
No	128 (38.09)	16 (36.36)	5 (19.23)	

*Constipation*				
Yes	286 (85.11)	40 (90.90)	17 (65.38)	**0.008** (7.04)
No	50 (14.88)	4 (9.09)	9 (34.61)	

*Asthenia*				
Yes	286 (85.11)	42 (95.45)	26 (100)	0.27 (1.21)
No	50 (14.88)	2 (4.54)	0 (0)	

*Type of stool*				
Mucoid	167 (49.70)	26 (59.09)	10 (38.46)	
Watery	78 (23.21)	11 (25.00)	6 (23.07)	
Hard	74 (22.02)	5 (11.36)	8 (30.76)	0.17 (4.97)
Bloody	17 (5.05)	2 (4.54)	2 (7.69)	

*Anorexia*				
Yes	303 (90.17)	28 (63.63)	23 (88.46)	
No	33 (9.82)	16 (36.36)	3 (11.53)	**0.02** (5.09)

*Abdominal pains*				
Yes	324 (96.42)	42 (95.45)	25 (96.15)	0.88 (0.019)
No	12 (3.57)	2 (4.54)	1 (3.84)	

^*∗*^Values in brackets are in percentage (%). In column three, the denominator is 44. In column four, the denominator is 26. Numbers in bold represent the *P* value of each sociodemographic characteristic which is significantly different compared to the normal *P* value (0.05).

**Table 4:** Multivariate analysis of sociodemographic characteristics of study participants.

Variable	Total (*N* = 336)	Typhoid fever positive (*N* = 70)	*p* value	OR (95% CI)
*Gender*				
Female	196 (58.33)	53 (27.17)	0.817	0.514 (0.445–1.501)
Male	140 (41.66)	17 (12.14)	—	1

*Age groups (years)*				
[1–10]	195 (58.03)	41 (21.02)	0.545	0.414 (0.017–10.328)
[11–20]	59 (17.55)	13 (22.03)	0.591	0.889 (0.041–19.431)
[21–30]	33 (9.82)	8 (24.24)	0.94	1.313 (0.063–27.580)
[31–40]	28 (8.33)	5 (17.85)	0.861	1.646 (0.084–32.179)
[41–50]	13 (3.86)	2 (15.38)	0.742	2.845 (0.105–77.173)
[51–60]	8 (2.38)	1 (12.50)	0.535	1

*Level of education*			—	
Primary	137 (40.77)	30 (15.30)	0.779	1.242 (0.274–5.621)
Secondary	153 (45.53)	30 (19.60)	0.578	1.533 (0.341–6.892)
Higher	22 (6.54)	6 (27.27)	0.959	1.053 (0.144–7.700)
No level	24 (7.14)	4 (16.66)	—	1

*Marital status*				
Married	61 (18.15)	16 (11.76)	0.454	0.441 (0.052–3.756)
Single	224 (66.66)	46 (30.06)	0.534	0.49 (0.052–4.643)
Divorced	32 (9.52)	5 (22.72)	0.991	0.988 (0.124–7.895)
Widow	19 (5.65)	3 (12.50)	—	1

*Occupation*				
Student	218 (64.88)	35 (16.05)	0.779	3.974 (0.420–37.576)
Private sector	94 (27.97)	30 (31.91)	0.578	0.418 (0.046–3.768)
Civil servant	12 (3.57)	2 (16.66)	0.959	1.076 (0.057–20.302)
House wife	12 (3.57)	3 (25)	—	1

*Residence*				
Urban area	216 (64.28)	46 (21.29)	0.730	1.135 (0.554–2.323)
Rural area	120 (35.71)	24 (20)	—	1

^*∗*^Values in brackets are in percentage (%). In this table, the denominator for column three corresponds to the total of each variable.

**Table 5:** Multivariable analysis of life style of study participants.

Life style	Total (*N* = 336)	Typhoid fever positive (*N* = 70)	*p* value	OR (95% CI)
*Do you know about typhoid fever?*				
Yes	328 (97.61)	66 (20.12)	**0.043**	5.243 (1.052–26.119)
No	8 (2.38)	4 (50)	—	1

*If yes, state knowledge source*				
Hospital	69 (20.53)	19 (27.53)	0.175	0.586 (0.271–1.269)
School	117 (34.82)	24 (20.51)	0.561	0.811 (0.401–1.642)
Media	27 (8.03)	6 (22.22)	0.968	1.023 (0.333–3.147)
Street	123 (36.60)	21 (17.07)	—	1

*Typhoid transmission route*				
Soiled food	174 (51.78)	38 (21.83)	0.303	2.026 (0.528–7.777)
Blood route	13 (3.86)	2 (15.38)	0.566	1.812 (0.238–13.802)
Contact with a sick person	41 (12.20)	10 (24.39)	0.276	2.335 (0.508–10.727)
Soiled water	96 (28.57)	17 (17.70)	0.099	3.311 (0.800–13.707)
From mother to child	12 (3.57)	3 (25)	—	1

*Hygiene measures against typhoid*				
Do not wash hands after bowel movements	86 (25.59)	11 (12.79)	**0.004**	3.740 (1.515–9.234)
Hand washing before and after meal	46 (13.69)	10 (21.73)	0.152	2.095 (0.762–5.761)
Washing fruits and vegetables before consumption	82 (24.40)	17 (20.73)	0.093	2.085 (0.883–4.922)
Hand washing after returning from public places	70 (20.83)	15 (21.42)	0.217	1.724 (0.726–4.093)
Washing hands after greeting a loved one	52 (15.47)	17 (32.69)	—	1

*Drinking water used*				
Camwater	82 (24.40)	22 (26.82)	0.192	0.326 (0.060–1.758)
Rain water	6 (1.78)	1 (16.66)	0.553	0.436 (0.028–6.783)
Well	21 (6.25)	7 (33.33)	0.156	0.258 (0.040–1.676)
River	14 (4.16)	4 (28.57)	0.193	0.258 (0.034–1.980)
Drilled holes (bore holes)	200 (59.52)	34 (17)	0.645	0.679 (0.132–3.510)
Mineral	13 (3.86)	2 (15.38)	—	1

*Hygiene measures used to make water safe to drink*				
Chlorination	48 (14.28)	8 (16.66)	0.104	2.104 (0.858–5.160)
Filtration	84 (25)	13 (15.47)	0.089	1.910 (0.906–4.031)
Boiling	13 (3.86)	2 (15.38)	0.182	3.148 (0.585–16.931)
Decanting	21 (6.25)	2 (9.52)	0.149	3.093 (0.668–14.327)
None	170 (50.59)	45 (26.47)	—	1

^*∗*^Values in brackets are in percentage (%). In column two, the denominator is 336. In column three, the denominator corresponds to the total of each variable in the lifestyle. Numbers in bold represent the *P* value of each lifestyle which is significantly different compared to the normal *P* value (0.05).

**Table 6:** Typhidot immunoassay readout for typhoid fever.

Tests	Observation	Number of patients	Percentage (%)
1	Both IgM and IgG positive	92	27.38
2	IgM positive and IgG negative	73	21.72
3	Both IgM and IgG negative	60	17.85
4	IgM positive	117	34.82
5	IgG positive	159	47.32

^*∗*^In this table, the denominator for column three is 336.

**Table 7:** Distribution of clinical symptoms among study participants.

Clinical symptoms	Total (*N* = 336)	Typhoid fever positive (*N* = 70)	*p* value	OR (95% CI)
*Headaches*				
Yes	232 (69.04)	49 (21.12)	0.365	0.739 (0.385–1421)
No	104 (30.952)	21 (20.19)	—	1

*Vomiting*				
Yes	189 (56.25)	35 (18.51)	0.28	1.373 (0.772–2.441)
No	147 (43.75)	35 (23.80)	—	1

*Aches and pain*				
Yes	234 (69.64)	48 (20.51)	0.651	1.157 (0.615–2.173)
No	102 (30.35)	22 (21.56)	—	1

*Fever*				
Yes	268 (79.76)	55 (20.52)	0.705	1.147 (0.565–2.328)
No	68 (20.23)	15 (22.05)	—	1

*Shivering*				
Yes	281 (83.63)	59 (20.99)	0.659	0.836 (0.377–1.853)
No	55 (16.36)	11 (20)	—	1

*Diarrhea*				
Yes	208 (61.90)	49 (23.55)	0.272	0.701 (0.371–1.322)
No	128 (38.09)	21 (16.40)	—	1

*Constipation*				
Yes	286 (85.11)	57 (19.93)	0.264	1.566 (0.713–3.438)
No	50 (14.88)	13 (26)	—	1

*Asthenia*				
Yes	286 (85.11)	68 (23.77)	0.233	0.366 (0.070–1.911)
No	50 (14.88)	2 (4)	—	1

*Type of stool*				
Mucoid	167 (49.70)	36 (21.55)	0.851	1.120 (0.344–3.643)
Watery	78 (23.21)	17 (21.79)	0.876	1.104 (0.319–3.826)
Hard	74 (22.02)	13 (17.56)	0.571	1.444 (0405–5.144)
Bloody	17 (5.05)	4 (23.52)	—	1

*Anorexia*				
Yes	303 (90.17)	51 (16.83)	**≤0.001**	7.703 (3.507–16.919)
No	33 (9.82)	19 (57.57)	—	1

*Abdominal pain*				
Yes	324 (96.42)	67 (20.67)	0.823	1.186 (0.265–5.302)
No	12 (3.57)	3 (25)	—	1

^*∗*^Values in brackets are in percentage (%), denominator column 2 : 336, and denominator column 3 corresponds to the total of each clinical symptoms. ^*∗*^Numbers in bold represent the *P* value of each clinical symptom which is significantly different compared to the normal *P* value (0.05).

**Table 8:** Validity of Widal and Typhidot immunoassays as diagnostic tools in comparison with stool culture for the diagnosis of typhoid fever.

Stool culture			*p* value	Sen	Spec	PPV	NPV	Accu	Kappa (95% CI)	Agreement (%)
	Pos	Neg								

*Typhidot-IgM*										
Pos	70	47		1	0.823	0.598	1	0.860	0.660	70.26
Neg	0	219	<0.0001	0.948–1.00	0.772–0.867	0.536–0.687	0.983–1.00	0.818–0.895	0.575–0.744	

*Widal*										
Pos	69	90		0.985	0.661	0.434	0.994	0.729	0.433	51.54
Neg	1	176	<0.0001	0.923–0.999	0.601–0.718	0.392–0.476	0.961–0.999	0.678–0.776	0.358–0.522	

PPV: positive predictive value; NPV: negative predictive value; Pos: positive; Neg: negative; Accu: accuracy; Sen: sensitivity; Spec: specificity; IgM: immunoglobulin M.

**Table 9:** Prevalence of typhoid fever based on Widal, Typhidot-IgM, and stool culture method.

Diagnostic test	Positive	Prevalence (%)	*p* value
Stool culture	70	20.86	
Widal	159	47.32	<0.0001
Typhidot	117	34.82	

## Data Availability

The datasets and analyzed data and materials used during the present study are available from the corresponding author upon reasonable request.
